# Out-of-pocket expenditure and distress financing on institutional delivery in India

**DOI:** 10.1186/s12939-019-1001-7

**Published:** 2019-06-25

**Authors:** Suyash Mishra, Sanjay K. Mohanty

**Affiliations:** 1 International Institute for Population Sciences, Govandi Station Road, Deonar, Mumbai, 400088 India; 2 Department of Fertility Studies, International Institute for Population Sciences, Govandi Station Road, Deonar, Mumbai, 400088 India

**Keywords:** Institutional delivery, India, OOPE, Coping strategy, Distress financing

## Abstract

**Background:**

Despite large investment in central and state sponsored schemes for maternal care, out-of-pocket expenditure (OOPE) and catastrophic health spending (CHS) on institutional delivery remain high over time, across states and across socio-economic groups. Though many studies have examined the OOPE and CHS, few studies have examined the nature and extent of distress financing on institutional delivery in India.

**Data:**

Data from the fourth round of National Family Health Survey (NFHS 4), 2015–16 was used for the analysis. Distress financing was defined as borrowing money or selling assets to meet the OOPE on delivery care. Composite variables, descriptive analyses, concentration index (CI), concentration curve (CC) and predicted probability were used to estimate the extent of distress financing for institutional delivery in India.

**Results:**

The OOPE on institutional delivery has strong economic and educational gradient. One in four mothers resorted to borrowing or selling to meet the OOPE on institutional delivery. The extent of distress financing on institutional delivery was high in poorer state of Bihar and Odisha and in the state of Telangana that had highest prevalence of caesarean delivery. Savings was more prevalent among mothers compared to those who met the OOPE by borrowing/selling of assets. Finding are robust across the states of India. The predicted probability of incurring distress financing was 0.31 among mothers belonging to the poorest wealth quintile compared to 0.09 in the richest quintile, and 0.40 for those who incurred OOPE of more than INR 20,000. The probability of incurring distress financing was higher for mothers who had caesarean birth, delivered in private health centers and incurred high OOPE on institutional delivery.

**Conclusion:**

Distress financing on institutional delivery was higher among the less educated, poor and in private health centers. Increasing use of public health centers, reducing caesarean births, improving the availability of medicine and diagnostic services can reduce the extent of distress financing in India.

## Introduction

High and increasing health care cost is one of the major public health challenges in developing countries. While the household remains the major source of financing health care, the extent of poverty, impoverishment and indebtedness due to high out-of-pocket expenditure (OOPE) is on the rise [1–4]. An estimated 808 million people across 133 countries are said to have incurred catastrophic health spending (CHS) [5]. CHS varies enormously across and within countries and is consistently high among the poor, less educated, uninsured, rural households, female headed households, households with members suffering from chronic illness and households with older people [6, 7]. Reduction of CHS has been integrated into global development agenda (SDGs) [8]. Besides CHS, high OOPE on medical care makes poor households poorer and drives non-poor household into poverty. About 97 million people in 2010 became impoverished ($1.90 per day poverty line) due to out of pocket health spending across 122 countries [9]. Households resort to multiple means to cope with the increasing OOPE on health care including current income, savings, selling of assets, borrowing from moneylenders and reduction in consumption expenditure. The adverse consequences of borrowing and selling assets to meet OOPE is profound in the short run as well as long run [10–13].

A number of studies coined borrowing and selling of assets by households as distress financing of health care [14–19]. Distress financing varies largely by the nature of the disease, type of facility used and the economic wellbeing of the household. The poor and marginalized are more likely to resort to distress financing across countries [17, 20, 21]. The causes of distress financing are many: high OOPE on healthcare including payments for medicines, consultation and procedure fees, low insurance coverage, financial constraints, and low government spending. Though recent literature has focused more on OOPE and CHS on health care, there is little emphasis on the source of meeting OOPE on health care. About 150 million people suffered from distress financing in seeking healthcare services [22]. OOPE on health care has an adverse impact on the economic wellbeing of individuals forcing them to resort to distress financing [3, 23–25]. The definition of distress financing is not uniform but is more context-specific [15, 17, 20, 26–29]. The extent, nature and correlates of distress financing for meeting health expenditure varies across countries [15, 16, 19, 27, 30, 31]. A number of studies from low and middle-income countries suggests that borrowing from relatives and friends, loans from money lenders and financial institutions, mortgaging assets, selling assets, selling livestock and selling harvest crops are common forms of distress financing [3, 25, 32–34]. Studies observe that other coping strategies to meet health care cost were reducing household food and non-food expenditure and increasing working hours for extra income [35]. Income diversification, selling of assets and borrowing money was common practice to meet the direct health care costs while task reallocation among household members was used for meeting the indirect costs of illness in low-income countries [36]. Poor households, households residing in rural areas, households suffering from multiple ailments and chronic diseases are more likely to incur distress financing [3, 17, 20, 29, 30, 37–40]. The borrowing and selling of assets was higher for treating tuberculosis and antiretroviral services as compared to obstetric care in South Africa [31]. Studies from East European countries suggest that lower health status, lower income, and chronic illness increases the likelihood of distress financing [19]. A study by Adam & Ke (2008) found that about 30% of the households across 15 African countries met their health expenditure by borrowing or selling assets [41]. About 26% of the households in urban India met their health expenditure by borrowing from different sources and 5% depended on selling of assets and livestock [42].

A number of studies examined the extent, nature and correlates of distress financing on maternal care. In rural Bangladesh, about 40% of the households relied on loans, donations from friends or relatives, sale of assets or combination of all the sources to meet severe maternal morbidity and about half of the households were able to avoid catastrophic health spending because of the coping strategies [27]. A study from three African countries revealed that about one-third of the women relied on selling of assets and crops to pay for delivery care expenses [43]. About 17.4% of the women from the lowest quintile in Mumbai slums financed the maternal care expenditure by borrowing [44]. Modugu et al. 2012 found that distress financing was higher for caesarean-section deliveries and was associated with high OOPE [45].

The healthcare system in India is characterized by co-existence of public and private health centers, poor public health infrastructure, high health care costs and low insurance coverage. The poor quality of services at public health centers and low insurance coverage lead to increasing use of private health centers and high OOPE in India. OOPE as share of total health expenditure has remained high over time- 69.4% in 2004, 64.2% in 2014 and 62.6% in 2015 [46–48]. During the last one and half decades, the Government of India and the state government have launched a number of financial protection schemes to reduce OOPE and CHS on maternal care. The Janani Suraksha Yojana (JSY) and Janani Shishu Suraksha Karyakram (JSSK) are two such centrally funded schemes under the National Health Mission (NHM) that provide conditional cash incentives to the mothers. The JSY was initiated in 2005 under which mothers delivering at public health centers in Low Performing States (LPS) were entitled to INR 1400 in rural areas and INR 1000 in urban areas and INR 700 in rural areas and INR 600 in urban areas in High Performing States (HPS) (https://www.nhp.gov.in/janani-suraksha-yojana-jsy-_pg). Introduced in 2011, the JSSK provides free and cashless services to pregnant women for both normal and caesarean deliveries and new-borns up to 30 days in Government health institutions. The Rashtriya Swasthya Bima Yojana (RSBY) was launched by the Government of India in 2008 with the objective of protecting poor households from the financial hardship associated with hospitalization (http://www.rsby.gov.in/how_works.html). In 2018, the Government of India launched the Ayushman Bharat, a centrally sponsored National Health Protection Scheme that aims to provide an annual health cover of up to INR 5 lakh to nearly 10 crore vulnerable poor households. It covered secondary and tertiary treatment, from any public/private empanelled hospitals across the country (https://www.india.gov.in/spotlight/ayushman-bharat-national-health-protection-mission). Besides, there are various state specific Community Health Insurance (CHI). For example, *ACCORD*, providing health insurance cover to indigenous people in Tamil Nadu and MAMTA provides cash assistance for maternal care and child nutrition in Odisha. While studies on JSY suggest increase in institutional delivery and reduction in OOPE, RSBY did not yield the desired effect [49–51].

The state variations in epidemiological transition (ET) and level of development are large and associated with OOPE, CHS, per capita public health spending and distress financing. Among the 36 states and union territories in India, 9 are classified in low ET, 8 in medium ET and 19 in high ET [52]. The composite Index of Human Development varies from 0.625 in Kerala to 0.442 in Odisha in 2011 [53]. Among the major states of India, the CHS was highest in Kerala (37.2%) and lowest in Assam (8.9%) [54]. Small scale studies suggest that the extent of distress financing was high in poorer states [8, 11].

Distress financing and CHS are alternative ways to capture the vulnerability of high OOPE. While the CHS is being quantified by alternative methods and debated, the utility of distress financing is its simplicity to capture the high health care costs. Many studies have estimated the extent of distress financing in health care in India [20, 29, 33, 44, 55, 56], but few have quantified it for delivery care [27, 45]. However, no study has estimated the association of OOPE and coping strategy with delivery care in India. In this context, our study aims to estimate the extent of distress financing on delivery care and its correlates using recently held large scale population based data in India.

## Data and method

We have used the unit data from the fourth round of National Family Health Survey (NFHS 4) conducted during 2015–16. The NFHS 4 has the distinction of being the largest ever population-based survey (sample size, geographical coverage and content) on maternal and child health in India. In line with the Demographic Health Survey, it collected detailed information on demographic, socio-economic condition of the households, contraception, utilisation of health services and nutritional status of children and mothers. The NFHS 4 has successfully interviewed a total of 601,509 households and 699,686 ever married women in 15–49 age group. A total of 259,627 births were reported in the 5 year preceding the survey, of which 190,898 were of last births and 148,746 were conducted at a health center. The instrument used, results of the survey along with methodology and sampling design is available in the national report [57].

For the first time, NFHS 4 collected information on OOPE on delivery care (defined as the expenditure net of reimbursement) for last birth that was delivered at a health center. Along with data on OOPE, the survey also provides information on source of financing for OOPE on delivery care. The unit data was cleaned for reported errors on OOPE before the analysis. The details and procedure of data cleaning is available elsewhere [58].

### Outcome variable

Distress financing is the outcome variable used in the analyses. A mother was said to be incurring distressed financing if she reported borrowing money or selling assets or reported utilising savings along with borrowing money or selling assets for meeting OOPE on institutional delivery.

### Independent variable

A number of independent variables were used in the analyses. These include: type of delivery, (normal / caesarean), source of delivery care (public/private),^1^ OOPE on delivery care, JSY beneficiary, and individual/household characteristics. The OOPE on institutional delivery was grouped into six categories; less than 1000, 1000–5000, 5000–10,000, 10,000–15,000, 15,000–20,000 and higher than 20,000. The individual characteristics include mothers’ age (15–24, 25–34, 35+), education (no education, primary, secondary, higher), parity (1, 2, 3, 4+), religion (Hindu, Muslim, Others), social group (Scheduled Castes [SCs] Scheduled Tribe [STs], Other Backward Classes [OBCs] and Others). Wealth quintile (poorest, poorer, middle, richer, richest)^2^ is used to reflect the economic status of the households .

### Statistical analysis

Descriptive statistics, composite variables, concentration index (CI), concentration curve (CC) and logistic regression were used in the analyses. To understand distress financing, a composite variable based on various source of financing was computed and categorized into five distinct groups; mothers those (i) used only their savings (ii) either borrowed money or sold assets (iii) used savings and either borrowed money or sold assets (iv) relied on insurance or other sources of financing and (v) did not pay.

The CC and CI were used to discern the economic inequality (measured by wealth index) in distress financing. The CC and CI are commonly used measure in health inequality research [59]. The CC graphically represents the economic inequality and plots the cumulative proportions of the population (ranked by wealth) against the cumulative proportions of population incurring distress financing. Thus, if the extent distress financing was evenly distributed across the wealth group, then the CC would coincide with the line of equality. However if CC lies above the line of equality, suggesting the concentration of distress financing among mother belonging to poor households while if CC lies below the line of equality, it implies concentration of distress financing among mothers belonging to rich households. The CI is derived from CC and is defined as twice the area between the concentration curve and line of equality, ranging from − 1 to + 1. The closer the CI value to 1 (absolute), the more unequal is distress financing across the wealth group while a zero value of CI suggest equal distribution of distress financing across the wealth group. The logistic regression (state fixed effect model) was used to predict the probability of distress financing on delivery care. The outcome variable has been coded as 1 if the mother incurred distress financing and 0 otherwise. The general form of the regression model is given as:

$$ {\displaystyle \begin{array}{l}\mathrm{logit}\kern0.5em \left({\uppi}_{\mathrm{i}}\right)\kern0.5em =\kern0.5em \upalpha \kern0.5em +\kern0.5em {\upbeta}_1\kern0.5em \left(\mathrm{place}\ {\mathrm{of}\ \mathrm{residence}}_{\mathrm{i}}\right)\kern0.5em +\kern0.5em {\upbeta}_2\left({\mathrm{age}}_{\mathrm{i}}\right)\kern0.5em +\kern0.5em {\upbeta}_3\left({\mathrm{e}\mathrm{ducation}}_{\mathrm{i}}\right)\kern0.5em +\kern0.5em {\upbeta}_4\left({\mathrm{religion}}_{\mathrm{i}}\right)\kern0.5em +\kern0.5em \\ {}{\upbeta}_5\left({\mathrm{social}\ \mathrm{group}}_{\mathrm{i}}\right)\kern0.5em +\kern0.5em {\upbeta}_6\left({\mathrm{birth}\ \mathrm{order}}_{\mathrm{i}}\right)\kern0.5em +\kern0.5em {\upbeta}_7\left({\mathrm{wealth}\ \mathrm{quintile}}_{\mathrm{i}}\right)\kern0.5em +\kern0.5em {\upbeta}_8\left(\mathrm{source}\ {\mathrm{of}\ \mathrm{delivery}}_{\mathrm{i}}\right)\kern0.5em +\kern0.5em \\ {}{\upbeta}_9\left(\mathrm{type}\ {\mathrm{of}\ \mathrm{delivery}}_{\mathrm{i}}\right)\kern0.5em +\kern0.5em {\upbeta}_{10}\left(\mathrm{JSY}\ {\mathrm{beneficiary}}_{\mathrm{i}}\right)\kern0.5em +\kern0.5em {\upbeta}_{11}\left({\mathrm{OOPE}}_{\mathrm{i}}\right)+{\mathrm{e}}_{\mathrm{i}}\end{array}} $$

where π_*i*_ is the probability of incurring distress financing by the i^th^ women on institutional delivery, α is the intercept and β‘s are the slope parameter. Results of the regression analyses are presented with the help of predicted probabilities. The analysis was restricted only to those states which had a minimum sample of 200 mothers.

## Results

### OOPE by wealth tertile and educational attainment

Table 1 presents the mean OOPE by wealth tertile and educational attainment of mothers in the states of India. In general, OOPE increases with wealth tertile suggesting that OOPE is associated with the households’ ability to pay. The mean OOPE among mothers belonging to the poorest wealth tertile was 2.5 times higher than that of the richer tertile. The pattern is similar with respect to educational attainment. Besides, OOPE on institutional birth varies enormously across the states of India. It was lowest in Madhya Pradesh (INR 4150) followed by Bihar (INR 4765) and highest in Kerala (INR 16149) followed by Delhi (INR 14960) and Manipur (INR 14822). In general, the OOPE was higher in economically better off states and low in the poorer states of India. The OOPE was higher in Telangana, which has recorded the highest caesarean deliveries in India. Similarly, the mean OOPE of an institutional birth of mothers with no education was INR 4382 compared to INR 9703 those with more than 7 years of schooling. The ratio of OOPE among mothers with 7 years of schooling and more to illiterate mothers was highest in Assam (3.53) and least in Delhi (0.49). The OOPE increases with wealth tertile and educational attainment, thereby suggesting that the richer and educated parents might be seeking better quality of care and care from private health centers.

**Table 1:** Mean OOPE on Institutional Delivery by Wealth Tertile and Educational Attainment in the States of India, 2015–16

State		Mean OOPE (INR)	Mean OOPE (INR)			Mean OOPE (INR)			N
			Wealth Tertile			Education			
			Poor	Middle	Rich	Illiterate	1–6 years	7+ years	
Andhra Pradesh		7946	5258	7395	9513	5927	6585	8920	5964
Assam		6121	3590	5789	14,361	2196	3557	7747	3713
Bihar		4765	3834	5843	9156	3393	4454	6639	13,667
Chhattisgarh		5278	2474	4182	10,280	1991	3363	6882	3216
Gujarat		8742	4801	6617	11,337	5335	7624	10,009	7306
Haryana		6643	4682	3243	7557	3896	4911	7583	3439
Himachal Pradesh		6027	4321	3741	6715	3650	3736	6217	685
Jammu and Kashmir		5371	3817	4285	6684	3946	4013	6164	1467
Jharkhand		5869	3712	7506	11,031	3011	5213	7387	3478
Karnataka		10,197	5412	7486	14,158	4553	6806	11,807	7412
Kerala		16,149	4887	10,217	17,177	5837	10,067	16,261	3659
Madhya Pradesh		4150	2127	3207	7644	2064	2626	5778	9898
Maharashtra		9838	6230	8203	12,110	6425	6918	10,620	14,355
Manipur		14,822	11,563	13,751	18,041	12,587	12,978	15,202	282
Meghalaya		6770	4515	6300	11,405	3885	4432	8447	291
Delhi		14,960	19,934	10,139	15,686	27,937	6890	13,722	2035
Odisha		5337	3964	5548	9412	2814	4092	6593	5750
Punjab		8488	3013	4433	9229	4027	5226	9696	2976
Rajasthan		5573	3948	5435	7240	4213	5195	6847	8860
Tamil Nadu		10,063	4440	6628	13,615	4334	5945	10,931	10,792
Tripura		6344	4589	7383	9353	3257	4500	7188	424
Uttar Pradesh		6747	3932	6796	11,056	4342	5145	8795	21,436
Uttarakhand		7735	4251	4700	9862	5666	5847	8479	1059
West Bengal		11,005	8985	12,360	13,495	5995	10,304	12,220	10,890
Telangana		13,567	11,059	11,962	15,247	11,011	11,776	14,496	4771
India		7978	4578	7154	11,527	4382	5976	9703	148,746

### Source of financing for institutional delivery

**Fig. 1:**
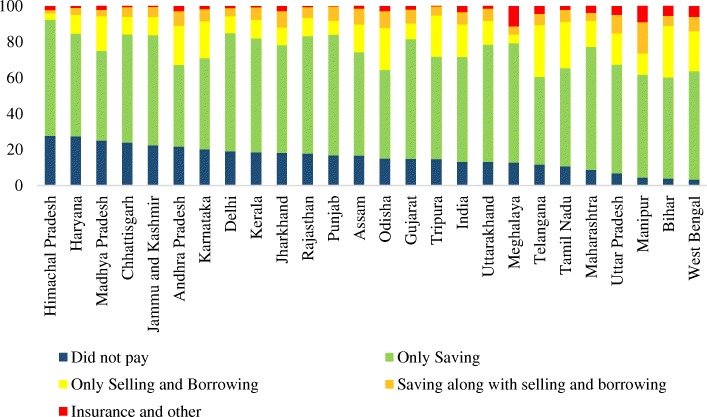
Source of Financing for Institutional Delivery in the States of India, 2015–16

Figure 1 shows the distribution of source of financing for institutional delivery across selected states of India. The state variation in source of financing for delivery care is large. Among the states, about 28% of the mothers in Himachal Pradesh and 27% of the mothers from Haryana did not pay for institutional delivery while it was lowest in West Bengal (3.2%) and Bihar (3.9%). It was 25% in Madhya Pradesh, 24% in Chhattisgarh and less than 7% in Uttar Pradesh. Over 60% of the mothers in the states of Punjab, Maharashtra, Gujarat, Meghalaya, Delhi, Rajasthan, Uttarakhand, Himanchal Pradesh, Kerala, Jammu and Kashmir, West Bengal, Uttar Pradesh, Chhattisgarh and Jharkhand paid from their savings. The proportion of mothers who met their expenses on institutional delivery by borrowing or selling assets was highest in the states of Telangana (29%) and Bihar (28.8%) followed by Tamil Nadu (26%) and Odisha (23.4%). About half of the women in Telangana had caesarean delivery and the cost of a caesarean delivery was at least three times higher than that of vaginal delivery [58]. Those who met the expenses on institutional delivery through combined means of saving, selling and borrowing was highest in Manipur (17.4%) followed by Uttar Pradesh (10.2%). From the analyses it is clear that the extent of distress financing on institutional delivery varies largely across the states of India.

Table 2 presents the mean OOPE on institutional delivery by source of financing across the states of India. The mean OOPE for mothers resorting to only saving for institutional delivery was highest in Kerala (INR. 19,076) followed by Delhi (INR 18994) and Manipur (INR 14480), while it was lowest in Bihar (INR 4271) followed by Madhya Pradesh (INR 5227). The mean OOPE for mothers relying on only selling and borrowing was highest in Kerala (INR 20621) followed by Telangana (INR 17618) and Manipur (INR 15625) while it was lowest in Assam (INR 4138) followed by Meghalaya (INR 5034). It was highest in Kerala for those who met the expenses on institutional delivery through savings along with selling and borrowing (INR 25994) followed by Delhi (INR 22340) and Karnataka (INR 21374). In the poorer states of Bihar and Uttar Pradesh, the mean OOPE among mothers who met the expenses on institutional delivery by selling or borrowing only was INR 5585 and INR 6788 rupees respectively. Results show that the OOPE was similar for those who met delivery expenses with savings or with borrowing/selling, but at least twice higher among those who met it through savings along with borrowing and selling. This further confirms that high OOPE on institutional delivery is leading to borrowing and selling assets.

**Table 2:** Mean OOPE (INR) (95% Confidence Interval) by Source of Financing in the States of India, 2015–16

State	Saving only	Confidence Interval	Only Selling and borrowing	Confidence Interval	Saving along with selling and borrowing	Confidence Interval	Insurance and other	Confidence Interval
Andhra Pradesh	7870	(7262, 8479)	13,158	(11,914, 14,403)	14,575	(12,320, 16,830)	10,582	(8325, 12,839)
Assam	7600	(7187, 8014)	4186	(3717, 4655)	10,592	(9139, 12,046)	10,655	(5486, 15,824)
Bihar	4271	(4077, 4465)	5585	(5061, 6109)	8780	(7868, 9692)	4885	(4161, 5609)
Chhattisgarh	6399	(5797, 7000)	5687	(4659, 6715)	14,774	(12,583, 16,965)	12,332	(7804, 16,861)
Delhi	18,944	(15,736, 22,152)	13,798	(8220, 19,376)	22,340	(15,276, 29,403)	15,366	(3734, 26,998)
Gujarat	9703	(9201, 10,206)	9482	(8400, 10,565)	16,024	(14,727, 17,321)	10,849	(8627, 13,071)
Haryana	8809	(8200, 9419)	8832	(7552, 10,113)	14,253	(11,970, 16,536)	12,913	(6925, 18,901)
Himachal Pradesh	7681	(6969, 8394)	13,312	(8070, 18,553)	20,426	(13,283, 27,568)	9589	(5565, 13,612)
Jammu and Kashmir	6731	(6380, 7082)	6907	(6019, 7795)	8970	(7269, 10,670)	6925	(5211, 8638)
Jharkhand	5543	(5113, 5972)	7709	(6600, 8818)	16,917	(15,308, 18,527)	8630	(6252, 11,009)
Karnataka	11,805	(11,169, 12,441)	11,792	(10,516, 13,068)	21,374	(18,716, 24,032)	18,842	(12,802, 24,882)
Kerala	19,076	(18,014, 20,137)	20,621	(176,565, 23,677)	25,994	(22,537, 29,451)	17,603	(11,752, 23,455)
Madhya Pradesh	5227	(4881, 5574)	5113	(4543, 5682)	12,665	(11,167, 14,163)	4541	(3104, 5979)
Maharashtra	10,445	(9909, 10,982)	10,124	(8396, 11,853)	14,425	(12,637, 16,212)	15,044	(10,055, 20,032)
Manipur	14,480	(13,857, 15,103)	15,625	(13,994, 17,257)	18,046	(16,785, 19,307)	16,759	(14,752, 18,765)
Meghalaya	8587	(7831, 9342)	5034	(3577, 6491)	5460	(3716, 7205)	5031	(3361, 6700)
Odisha	5782	(2488, 6075)	5905	(5443, 6368)	9520	(8509, 10,531)	7232	(5739, 8725)
Punjab	9122	(8264, 9620)	12,382	(10,591, 14,171)	17,157	(15,254, 19,060)	6192	(277, 12,107)
Rajasthan	6438	(5783, 7093)	5456	(4863, 6050)	13,041	(11,800, 14,282)	6087	(3972, 8202)
Tamil Nadu	9484	(8961, 10,006)	12,591	(11,743, 13,440)	19,804	(17,579, 22,029)	14,283	(10,846, 17,719)
Tripura	7681	(6955, 8408)	5837	(5183, 6492)	10,829	(8129, 13,528)	17,514	(−32,309, 67,336)
Uttar Pradesh	6361	(6119, 6602)	6788	(6248, 7328)	13,754	(12,916, 14,592)	6166	(5068, 7264)
Uttarakhand	7724	(7227, 8221)	9843	(7972, 11,714)	17,280	(14,571, 19,989)	14,068	(6912, 21,225)
West Bengal	13,906	(11,856, 15,957)	6172	(5427, 6918)	11,015	(9824, 12,207)	6596	(5196, 7997)
Telangana	13,847	(12,619, 15,076)	17,618	(16,010, 19,227)	20,609	(17,530, 23,687)	9830	(7497, 12,164)
India	8739	(8599, 8878)	8731	(8511, 8950)	14,542	(14,195, 14,888)	8708	(8124, 9293)

Table 3 shows the extent of distress financing by selected socio-demographic characteristics in India, Bihar and Telangana. The extent of distress financing was highest in Telangana followed by Bihar and so these two states are used for illustration. The extent of distress financing was higher in rural areas compared to urban areas, higher among mothers with low educational attainment and belonging to the poorest and poorer wealth quintiles. About 31.8% of the mothers with no education in India met OOPE on institutional delivery through distress financing compared to 17.3% among those with higher secondary education and above. Similarly, about 41.8% of the others belonging to the poorest wealth quintile in Bihar incurred distress financing compared to 10.7% among the richest wealth quintile in the state. The extent of distress financing was higher among mothers belonging to socially disadvantaged groups (SC/ST) compared to mothers belonging to others group. Mothers using private health centers for institutional delivery reported high distress financing compared to mothers using public health centers (27.8% vs. 23.4%). Mothers experiencing caesarean delivery reported higher distress financing compared to mothers having normal delivery (30.9% vs. 23.1%). The extent of distress financing also increases with OOPE. About 12.5% of the mothers who had an OOPE of less than INR 1000 financed incurred distress financing compared to 34.9% of the mothers who spent INR 20000 and more.

**Table 3:** Distress Financing by Selected Socio-demographic Characteristics in India, Bihar and Telangana, 2015–16

	India		Bihar		Telangana	
Variables	Distress Financing (%)	Did not pay (%)	Distress Financing (%)	Did not pay (%)	Distress Financing (%)	Did not pay (%)
Place of Residence						
Urban	20.8	12.3	22.1	3.1	27.6	12.6
Rural	27.0	13.6	35.9	4.0	42.5	10.7
Age						
15–24	26.0	13.6	33.3	4.0	36.7	11.1
25–35	24.2	12.9	34.4	3.7	34.3	11.8
35+	28.8	12.9	42.4	5.7	16.4	30.5
Education Level						
Illiterate	31.8	14.1	39.8	4.0	44.8	10.0
Primary	29.0	13.8	35.6	3.6	46.7	11.4
Secondary	25.0	13.4	30.8	3.1	35.9	12.1
Higher	17.3	11.7	19.9	4.8	26.5	12.0
Religion						
Hindu	24.9	13.3	33.5	3.9	36.3	11.3
Muslim	26.5	11.8	38.3	3.7	25.4	14.1
Other	21.1	14.4	65.9	0.0	37.2	11.3
Social Group						
Schedule caste	27.9	13.1	34.3	4.9	34.4	13.9
Schedule tribe	24.5	20.0	42.1	4.7	49.0	6.7
OBC	25.9	12.7	34.9	3.3	36.5	10.8
General	21.1	11.6	29.8	4.5	24.8	14.3
Birth Order						
1	23.9	12.9	33.6	4.1	33.3	14.6
2	24.2	13.1	32.0	3.5	35.4	9.7
3	26.0	13.9	35.6	3.5	40.4	11.1
4+	29.0	13.1	36.1	4.3	29.8	12.5
Wealth Quintile						
Poorest	35.1	12.7	41.8	4.0	47.3	9.2
Poorer	29.6	13.3	32.2	3.6	40.3	10.8
Middle	26.2	13.7	25.2	4.3	43.2	10.1
Richer	21.8	13.1	20.2	3.0	34.3	12.0
Richest	13.1	12.8	10.7	3.3	21.4	13.9
Source of Delivery						
Public health facility	23.4	16.5	34.3	3.6	27.2	13.9
Private health facility	27.8	7.0	33.8	4.5	39.0	10.5
Type of Delivery						
Normal delivery	23.1	14.5	33.8	3.9	26.7	12.1
Caesarean delivery	30.9	8.8	37.5	3.4	39.8	11.4
JSY Beneficiary						
No	25.5	11.2	35.9	3.5	36.9	10.4
Yes	24.2	15.6	33.0	3.3	23.6	16.6
Mean OOPE						
Less than 1000	12.5	na	25.7	na	11.5	na
1000–5000	28.4	na	36.4	na	26.5	na
5000–10,000	30.6	na	37.3	na	39.8	na
10,000–15,000	31.5	na	43.9	na	47.8	na
15,000–20,000	32.6	na	42.2	na	52.9	na
More than 20,000	34.9	na	44.7	na	46.7	na

**Fig. 2:**
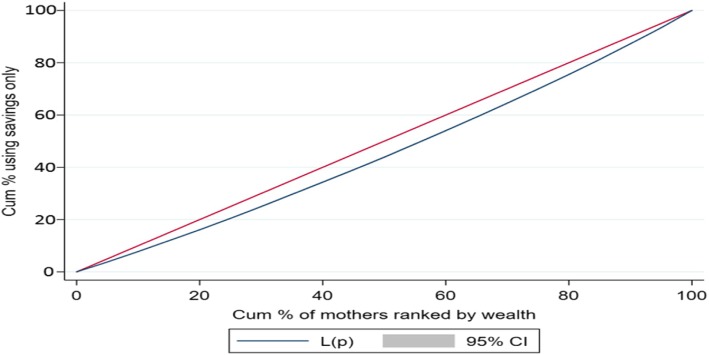
Concentration Curve for those meeting OOPE only by Savings in India, 2015–16

**Fig. 3:**
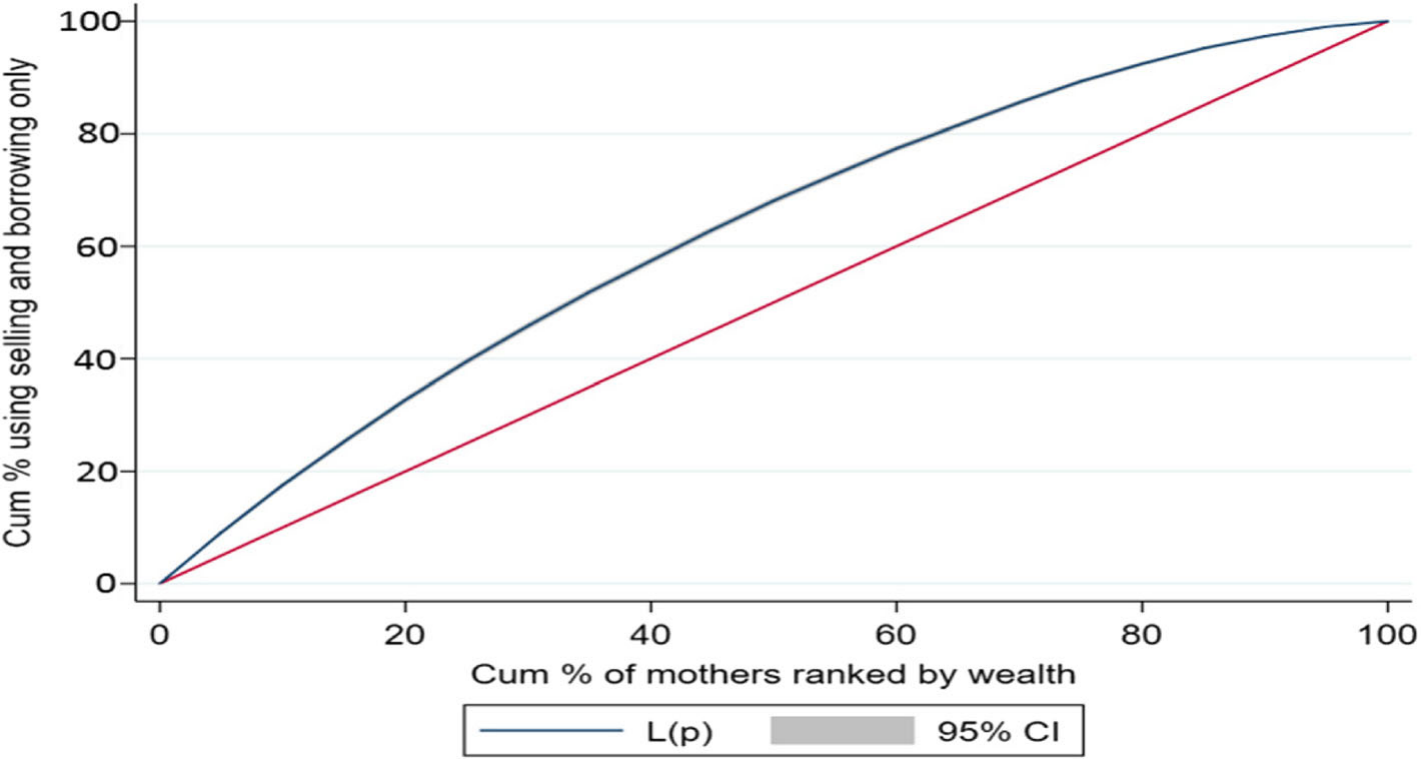
Concentration Curves for those meeting OOPE by only Selling and Borrowing in India, 2015–16

**Fig. 4:**
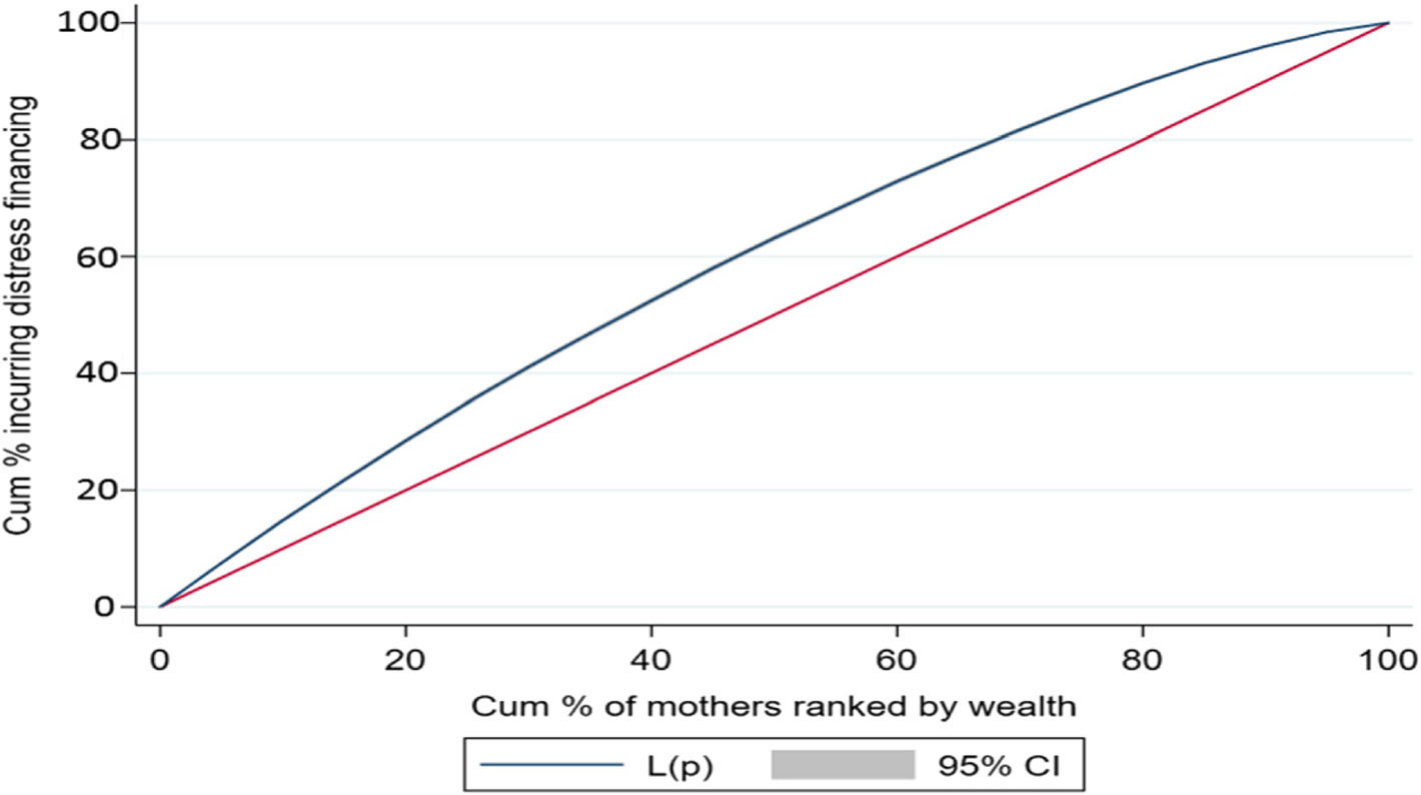
Concentration Curves for Incurring Distress Financing for Institutional delivery in India, 2015–16

Table 4 presents the concentration index for institutional delivery by source of financing in selected states of India. We present the CI for those mothers who met OOPE on institutional delivery with only saving, only by selling and borrowing, savings along with selling and borrowing and distress financing (any form of selling or borrowing). The CI value for mothers using only savings to meet OOPE on institutional delivery was 0.084 for India, and positive for all the states of India suggesting that savings was largely used to meet the OOPE among mothers belonging to rich households. At state level, the CI value of saving was highest in Madhya Pradesh (0.131) followed by Manipur (0.124) and least in Andhra Pradesh (0.028). Further the CI values for mothers using only selling and borrowing for institutional delivery was − 0.235 for India and was negative for all the states indicating that selling and borrowing only was largely used among mothers belonging to poor households. Similarly CI value for mothers incurring distress financing was − 0.171 for India and was negative for all the states. For instance, the CI value for mothers using only selling and borrowing was highest in Himachal Pradesh (− 0.442) followed by Meghalaya (− 0.416) and least for Andhra Pradesh (− 0.081) whereas the CI value for mother incurring distress financing for institutional delivery was highest in Kerala (− 0.316) followed by Jammu & Kashmir (− 0.312) and least in Karnataka (− 0.052). Figure 2 presents the CC for mother meeting OOPE through savings only, Fig. 3 presents the CC for mothers meeting the OOPE through selling and borrowing only and Fig. 4 presents the CC for mothers incurring distress financing for institutional delivery. The CC for mother using only savings to meet OOPE for institutional delivery was below the line of equality suggesting higher concentration of use of savings among mothers belonging to rich households. The CC for mothers using only selling and borrowing and incurring distress financing was above the line equality suggesting that the use of selling and borrowing and incurring distress financing among mothers belonging to poor households.

**Table 4:** Concentration Index for institutional Delivery by Source of Financing in Selected States of India, 2015–16

State	Only Saving	Only Selling and Borrowing	Savings along with Selling and Borrowing	Distress Financing
Andhra Pradesh	0.028	−0.081	0.011	−0.057
Assam	0.118	−0.308	−0.06	− 0.218
Bihar	0.110	−0.185	0.035	−0.150
Chhattisgarh	0.109	−0.294	0.111	−0.154
Delhi	0.065	−0.390	− 0.130	− 0.305
Gujarat	0.094	−0.323	− 0.03	− 0.189
Haryana	0.121	−0.353	− 0.037	− 0.271
Himachal Pradesh	0.028	−0.442	− 0.050	− 0.306
Jammu and Kashmir	0.077	−0.389	− 0.172	− 0.312
Jharkhand	0.057	−0.209	0.119	−0.053
Karnataka	0.072	− 0.118	0.153	−0.052
Kerala	0.073	−0.353	− 0.257	− 0.316
Madhya Pradesh	0.131	−0.196	0.088	−0.152
Maharashtra	0.073	−0.206	− 0.049	− 0.171
Manipur	0.124	−0.263	− 0.236	− 0.247
Meghalaya	0.108	−0.416	− 0.087	− 0.263
Odisha	0.113	−0.232	− 0.024	− 0.173
Punjab	0.095	−0.366	− 0.042	− 0.201
Rajasthan	0.037	−0.227	0.006	−0.145
Tamil Nadu	0.071	−0.143	0.170	−0.080
Telangana	0.115	−0.194	0.067	−0.148
Tripura	0.068	− 0.187	−0.043	− 0.161
Uttar Pradesh	0.079	−0.258	0.037	−0.149
Uttarakhand	0.067	−0.286	0.029	−0.179
West Bengal	0.111	−0.276	0.034	−0.194
India	0.084	−0.235	0.000	−0.171

Table 5 presents the predicted probability of incurring distress financing on institutional delivery adjusting for socio-economic and demographic characteristics of mothers and households in India, Bihar and Telangana. Results suggests that place of residence, mother’s education, social group, wealth quintile, type of delivery and out-of-pocket expenditure are significant predictors of distress financing in India. The predicted probability of incurring distress financing among mothers residing in rural and urban India (0.19) was similar. For illiterate mothers, the predicted probability of incurring distress financing was 0.23 compared to 0.14 for mothers with higher education level. The predictive probability of incurring distress financing has a strong economic gradient- 0.31 for mothers belonging to the poorest wealth quintile, 0.26 for poorer, 0.20 for middle, 0.15 for richer and 0.09 for the richest wealth quintile. The predicted probability of incurring distress financing for mothers having caesarean birth was 0.21 compared to 0.18 for normal delivery. For mothers who incurred OOPE of more than INR 20000 on institutional delivery (.41), the predicted probability of incurring distress financing was approximately five times higher than mothers who incurred OOPE of less than INR 1000 (0.09). The state pattern was similar in many of these covariates.

**Table 5:** Predicted probabilities of Incurring Distress Financing on Institutional Delivery by Selected Socio-Demographic Characteristics in India, Bihar and Telangana, 2015–16

	India		Bihar		Telangana	
Variables	Predictive Probability	95% Confidence Interval	Predictive Probability	95% Confidence Interval	Predictive Probability	95% Confidence Interval
Place of Residence						
Urban	0.194	(0.189, 0.199)	0.273	(0.245, 0.302)	0.355	(0.306, 0.404)
Rural	0.186	(0.183, 0.189)	0.321	(0.311, 0.330)	0.357	(0.322, 0.393)
Mother Age						
15–24	0.195	(0.191, 0.199)	0.309	(0.293, 0.326)	0.365	(0.324, 0.407)
25–35	0.185	(0.182, 0.187)	0.316	(0.304, 0.329)	0.351	(0.315, 0.387)
35+	0.193	(0.180, 0.207)	0.355	(0.299, 0.411)	0.249	(−0.029, 0.527)
Education Level						
No education	0.229	(0.223, 0.234)	0.348	(0.334, 0.363)	0.418	(0.350, 0.486)
Primary	0.216	(0.210, 0.222)	0.320	(0.295, 0.346)	0.501	(0.415, 0.587)
Secondary	0.192	(0.189, 0.196)	0.303	(0.285, 0.321)	0.358	(0.318, 0.399)
Higher	0.140	(0.136, 0.144)	0.230	(0.206, 0.255)	0.271	(0.224, 0.318)
Religion						
Hindu	0.186	(0.183, 0.188)	0.309	(0.299, 0.319)	0.361	(0.332, 0.390)
Muslim	0.211	(0.205, 0.217)	0.350	(0.325, 0.376)	0.325	(0.241, 0.409)
Others	0.177	(0.168, 0.186)	0.651	(0.339, 0.963)	0.343	(0.191, 0.496)
Social Group						
Schedule caste	0.200	(0.195, 0.205)	0.298	(0.279, 0.318)	0.339	(0.280, 0.398)
Schedule tribe	0.190	(0.184, 0.196)	0.337	(0.284, 0.389)	0.400	(0.309, 0.491)
OBC	0.190	(0.187, 0.194)	0.325	(0.313, 0.337)	0.355	(0.320, 0.389)
Others	0.175	(0.170, 0.179)	0.294	(0.271, 0.317)	0.361	(0.287, 0.436)
Birth Order						
1	0.183	(0.179, 0.187)	0.323	(0.303, 0.342)	0.339	(0.294, 0.384)
2	0.187	(0.184, 0.191)	0.312	(0.295, 0.329)	0.364	(0.326, 0.402)
3	0.191	(0.185, 0.196)	0.321	(0.302, 0.341)	0.374	(0.303, 0.444)
4+	0.203	(0.196, 0.209)	0.304	(0.284, 0.324)	0.353	(0.215, 0.492)
Source of Delivery						
Public facility	0.188	(0.185, 0.191)	0.327	(0.315, 0.339)	0.327	(0.275, 0.378)
Private facility	0.188	(0.183, 0.194)	0.282	(0.258, 0.305)	0.373	(0.336, 0.411)
Type of Delivery						
Normal Delivery	0.183	(0.180, 0.185)	0.312	(0.302, 0.322)	0.317	(0.274, 0.360)
Caesarean Delivery	0.211	(0.206, 0.217)	0.339	(0.303, 0.374)	0.381	(0.346, 0.416)
Wealth Quintile						
Poorest	0.313	(0.305, 0.320)	0.394	(0.379, 0.409)	0.453	(0.341, 0.565)
Poorer	0.255	(0.249, 0.260)	0.306	(0.289, 0.323)	0.383	(0.318, 0.449)
Middle	0.201	(0.196, 0.205)	0.239	(0.217, 0.261)	0.406	(0.355, 0.457)
Richer	0.150	(0.146, 0.155)	0.191	(0.164, 0.219)	0.362	(0.314, 0.411)
Richest	0.084	(0.080, 0.087)	0.113	(0.077, 0.149)	0.222	(0.161, 0.282)
JSY Assistance						
No	0.189	(0.186, 0.192)	0.331	(0.314, 0.347)	0.359	(0.331, 0.388)
Yes	0.188	(0.184, 0.191)	0.303	(0.290, 0.316)	0.337	(0.257, 0.417)
Mean OOPE						
0–1000	0.085	(0.083, 0.088)	0.221	(0.207, 0.236)	0.137	(0.098, 0.175)
l000–5000	0.215	(0.211, 0.219)	0.316	(0.303, 0.329)	0.272	(0.216, 0.328)
5000–10,000	0.290	(0.282, 0.297)	0.429	(0.395, 0.463)	0.408	(0.339, 0.477)
10,000–15,000	0.328	(0.317, 0.339)	0.476	(0.424, 0.528)	0.476	(0.403, 0.549)
15,000–20,000	0.368	(0.352, 0.383)	0.503	(0.433, 0.573)	0.486	(0.389, 0.583)
20,000+	0.408	(0.396, 0.419)	0.560	(0.512, 0.607)	0.551	(0.495, 0.606)

## Discussion

Increasing institutional delivery and reduction of high OOPE and CHS on maternal care had received program priority since the implementation of the National Health Mission in India. In last decade, over half of the national health budget is spent on maternal care, and the state governments supplement to the national spending of the respective states. The JSY, under NHM is one the largest ever cash assistance programme worldwide. The NHM had been successful in reduction of maternal and child mortality and increasing maternal care, but studies suggest that the extent of OOPE and CHS continue to be high on maternal care [23, 60]. People continue to resort to different means such as selling assets, selling property and borrowing with and without interest to meet the expenses on delivery care. Studies examined the extent of distress financing on health care in India and a few have quantified it to delivery care [20, 45]. For the first time, the NFHS 4 provides information on out-of-pocket expenditure as well as source of meeting the delivery care expenses along with other maternal, child and household characteristics. In this paper, we have estimated the extent of distress financing and examined the correlates of distress financing in India using NFHS 4 data. We present the salient findings and provide plausible explanation of our findings.

First, the OOPE on institutional delivery has a strong economic and educational gradient and the findings are robust across the states of India. The OOPE on delivery care was high in private health centers, for pregnancy complications and caesarean births.. The state variation in OOPE on institutional delivery was large- higher in economically better off states and lower in the poorer states of India. This finding is consistent with literature [45, 58, 61]. Second, about two-fifths of the mothers utilized their savings, one-fifth of them resorted to only selling and borrowing, while one in seven mothers borrowed/sold assets in addition to using their savings to meet the OOPE on institutional delivery. The extent of distress financing was higher among the poorer, less educated mothers, in private health centers and for caesarean delivery. Only one in ten mothers did not pay for OOPE. Third, the OOPE on institutional delivery is positively associated with distress financing. While the OOPE on delivery was almost similar for those who used only saving and those who borrowed money or sold assets to meet the cost of delivery care, it was 70% higher for those who used savings in addition to borrowing or selling assets. Controlling for socio-economic correlates, the predicted probability of incurring distress financing was 0.41 among those spent INR 20,000 and above compared to 0.09 among those who spent less than INR 1000. Fourth, the state pattern of OOPE and distress financing is mixed. While only 13% of the mothers did not pay for institutional delivery, it was over 25% in Haryana, Himachal Pradesh and Madhya Pradesh and less than 10% in Bihar, Uttar Pradesh, West Bengal and Manipur. The extent of distress financing was also high in the states of Bihar, Telangana, Andhra Pradesh West Bengal and Odisha. It is interesting to note that the national average of OOPE among mothers who met OOPE by saving only and selling and borrowing only was almost similar while those met through savings along with borrowing and selling was approximately twice higher. Fifth, the CI value of distress financing for institutional delivery was −0.171 suggesting that the extent of distress financing is largely concentrated among the poor. The CI value was higher for mothers belonging to poor households who met health spending through borrowing and selling only (− 0.235). The state pattern of CI values for mothers who met OOPE through savings only and through saving along with selling or borrowing are robust across states of India. It further suggests that the inequality in meeting the OOPE through selling and borrowing was largely concentrated among the poor. These estimates are in expected directions.

We provide some plausible explanations in support of our results. Despite one and half decades of implementation of NHM that provides free delivery care in public health centers and entitles cash assistance, about one-fourth of the mothers resort to borrowing or selling asset to meet the OOPE on institutional delivery. This is possibly due to high OOPE resulting from increasing use of private health centers, increasing caesarean delivery and pregnancy complications. A recent study suggests that the OOPE of a caesarean delivery in a private health center was at least ten time that of a normal delivery [58]. Such high OOPE on delivery care might be leading to high distress financing. Those who utilized their savings in addition to borrowing and selling of assets paid almost twice the amount paid by those using saving only or selling and borrowing only and are more likely to face distress financing. Besides, the inter-state variation in OOPE and distress financing suggests that the provisioning of medicine, tests and other charges in public health centers vary across the states of India. Health is a state subject and the pattern of public spending varies largely across the state. It is found that the average spending in public health centers of low performing states is higher than that in high performing states. For example, in Tamil Nadu, patients seeking care from public health centers pay less on tests and medicine compared to patients in Bihar and Uttar Pradesh [62, 63]. Such variations might be leading to high OOPE and distress financing on delivery care. The extent of distress financing was higher in Telangana, Bihar and other poorer states in India. About half of the delivery in Telangana were caesarean confirming that high caesarean births lead to distress financing. Similarly, in the poorer states of Bihar and Uttar Pradesh, there is inadequate provisioning of medicine and diagnostics in public health centers that might be leading to high distress financing on institutional delivery [64]. The CI value of − 0.235 for those using selling and borrowing to meet the OOPE for institutional delivery suggests that the extent of distress financing is higher among mothers from poor households. This suggests that poor people are mostly affected due to high OOPE. Though the JSY and other state specific schemes are operational that provide financial assistance to the poor, the amount stipulated is not sufficient to meet the cost of delivery care. Thus, the high OOPE forces poor mothers to resort for alternative sources of financing in meeting the OOPE on institutional delivery. Even among mothers who received JSY assistance, one-fourth of the mothers incurred distress financing to manage the expenditure on institutional delivery. Thus not only the outreach of JSY remains low, the assistance provided is also insufficient to cover the expenditure incurred on institutional delivery.

In January 2017, the Government of India introduced *Pradhan Mantri Matru Vandana Yojana* (PMMVY) that stipulated partial compensation or the wage loss in the form of cash assistance of INR 5000 to pregnant and lactating mothers. A sum of INR 1000 is given on early registration of pregnancy at the Anganwadi Centre (AWC) / Approved Health facility, INR 2000 after 6 months of pregnancy on receiving at least one ante-natal check-up (ANC) and a sum of INR 2000 after child birth is registered and the child has received the first cycle of Bacillus Calmette Guerin (BCG), Oral Polio Vaccine (OPV), Diphtheria, Pertussis, Tetanus (DPT) and Hepatitis B, or its equivalent/ substitute. It aims at improving the health seeking behaviour amongst pregnant women and lactating mothers.

Although this study provides estimates of distress financing and its correlates of institutional delivery, it has certain limitations. First, we have not quantified the extent of borrowing and selling of assets due to data limitations. Data on the amount of borrowing and selling would have been helpful to understand the extent of indebtedness. Second, we could not assess the short run and long run indebted ness due to borrowing and selling of assets. Borrowing from money lenders at a high rate of interest affects the welfare of the household in the long run. Third, the OOPE incurred by mothers on delivery care may have recall bias and did not include the recent benefit under PMMVY. Despite these limitations, this paper provides comprehensive and robust estimates of distress financing in India.

## Conclusion

Our study highlights the presence of strong economic and educational gradient in OOPE associated with institutional delivery in India. Further, factors such as lower economic status, use of private health centres, having caesarean births increased the likelihood of OOPE on institutional delivery in India. Mothers resorted to various coping strategies to meet the OOPE on institutional delivery and hence reduction of distress financing requires reduction of OOPE on delivery care. This can be made possible by reducing the number of caesarean births whenever permissible, improving the services in public health centers, improving the availability of medicine and supplies, diagnostic services and effective implementation of maternity benefit schemes at national and state level. We further suggests that the coverage and benefits provided by various centrally sponsored schemes should be strengthened, monitored and regulated periodically. The state government has a greater role to play in the effective implementation of NHM. Also, population-based surveys should collect information on the amounts borrowed with or without interest and the value of assets sold to understand the degree of distress financing.

## Data Availability

The dataset used and analysed for the current study is available in DHS repository, [https://dhsprogram.com/data/dataset/India_Standard-DHS_2015.cfm?flag=0].

## Abbreviations

ANC: Ante-natal Checkup
AWC: Anganwadi Centre
BCG: Bacillus Calmette Guerin
CC: Concentration Curve
CHI: Community Health Insurance
CHS: Catastrophic Health Spending
CI: Concentration Index, Confidence Interval
DPT: Diphtheria, Pertussis, Tetanus
ET: Epidemiological Transition
HPS: High Performing States
JSSK: Janani Shishu Suraksha Karyakram
JSY: Janani Suraksha Yojana
LPS: Low Performing States
NFHS: National Family Health Survey
NGO: Non-governmental Organisation
NHM: National Health Mission
OBC: Other Backward Caste
OOPE: Out of Pocket Expenditure
OPV: Oral Polio Vaccine
PMMVY: Pradhan Mantri Matru Vandana Yojana
RSBY: Rashtriya Swasthya Bima Yojana
SC: Scheduled Caste
ST: Scheduled Tribe
UT: Union Territory

## Footnotes

1 Public source include government/municipal hospital, government dispensary, urban health centers, community health centers, sub-centers, rural hospital and other public health facilities while private source include hospitals, maternity home, clinic, non-governmental organization (NGO), trust hospital, clinic and other private sector facility.

2 Wealth quintile was derived from the wealth index that was created using Principal Component Analysis (PCA) for a set of 43 consumer durables for rural and urban areas separately. The consumer durables included source of drinking water, type of toilet facility, type of cooking fuel, main material of floor, main roof material and others household assets.
